# Identification of Lactic Acid Bacteria in Fruit Pulp Processing Byproducts and Potential Probiotic Properties of Selected *Lactobacillus* Strains

**DOI:** 10.3389/fmicb.2016.01371

**Published:** 2016-08-30

**Authors:** Estefânia F. Garcia, Winnie A. Luciano, Danilo E. Xavier, Whyara C. A. da Costa, Kleber de Sousa Oliveira, Octávio L. Franco, Marcos A. de Morais Júnior, Brígida T. L. Lucena, Renata C. Picão, Marciane Magnani, Maria Saarela, Evandro L. de Souza

**Affiliations:** ^1^Laboratório de Microbiologia de Alimentos, Departamento de Nutrição, Universidade Federal da ParaíbaJoão Pessoa, Brazil; ^2^Laboratório de Processos Microbianos em Alimentos, Departamento de Engenharia de Alimentos, Universidade Federal da ParaíbaJoão Pessoa, Brazil; ^3^Laboratório de Genômica e Proteômica, Universidade Católica de BrasíliaBrasília, Brazil; ^4^Grupo Interdepartamental de Pesquisa em Engenharia Metabólica, Departamento de Genética, Universidade Federal de PernambucoRecife, Brazil; ^5^Instituto de Microbiologia Paulo Góes, Universidade Federal do Rio de JaneiroRio de Janeiro, Brazil; ^6^VTT Technical Research Centre of FinlandEspoo, Finland

**Keywords:** fruit, 16S rRNA gene sequencing, MALDI-TOF profiling, *Lactobacillus*, probiotic

## Abstract

This study aimed to identify lactic acid bacteria (LAB) in byproducts of fruit (*Malpighia glabra* L., *Mangifera indica* L., *Annona muricata* L., and *Fragaria vesca* L.) pulp processing. Fifty strains of LAB were identified using matrix-assisted laser desorption/ionization–time of flight mass spectrometry (MALDI-TOF MS) and 16S rRNA gene sequence (16S rRNA) analysis. Species belonging to *Lactobacillus* genus were the predominant LAB in all fruit pulp processing byproducts. The average congruency between the MALDI-TOF MS and 16S rRNA in LAB species identification reached 86%. Isolates of *L. plantarum, L. brevis, L. pentosus, L. lactis* and *L. mesenteroides* were identified with 100% congruency. MALDI-TOF MS and 16S rRNA analysis presented 86 and 100% efficiency of LAB species identification, respectively. Further, five selected *Lactobacillus* strains (*L. brevis* 59, *L. pentosus* 129, *L. paracasei* 108, *L. plantarum* 49, and *L. fermentum* 111) were evaluated for desirable probiotic-related properties and growth behavior on two different cultivation media. The exposure to pH 2.0 sharply decreased the counts of the different *Lactobacillus* strains after a 1 or 2 h incubation, while varied decreases were noted after 3 h of exposure to pH 3.0. Overall, the exposure to pH 5.0 and to bile salts (0.15, 0.30, and 1.00%) did not decrease the counts of the *Lactobacillus* strains. All tested *Lactobacillus* strains presented inhibitory activity against *Staphylococcus aureus, Salmonella* Typhimurium, *Salmonella* Enteritidis, *Listeria monocytogenes* and *Escherichia coli*, and presented variable susceptibility to different antibiotics. The selected *Lactobacillus* strains presented satisfactory and reproducible growth behavior. In conclusion, MALDI-TOF MS and 16S rRNA analysis revealed high efficiency and congruency for LAB species identification, and the selected *Lactobacillus* strains may be candidates for further investigation of novel probiotic strains.

## Introduction

Consumption of fruit and fruit products (mostly low-processed juices and frozen pulps) has been increasing due to growing recognition of their nutritional value associated with their high content of minerals, vitamins, and secondary phytochemical compounds (Rufino et al., 2010; Silva et al., 2014). The processing of fruit generates a great amount of industrial byproducts, representing 10–60% of the total fruit weight (Ayala-Zavala et al., 2010). These byproducts comprise peels, rinds, seeds, and unused flesh, which are usually inappropriately discarded in the environment, leading to waste accumulation, and negative environmental impacts (Ajila et al., 2007; Araújo et al., 2014).

The economics of processing tropical fruit could be improved by developing higher value use for their byproducts (Silva et al., 2014). In addition to the known potential use of tropical fruit pulps and their byproducts for the isolation of phytochemicals for application in nutraceutical supplements (Ayala-Zavala et al., 2011), these byproducts also exhibit a wide variety of microorganisms of interest to the food industry (Yang et al., 2010), especially lactic acid bacteria (LAB). Each particular type of fruit provides a unique environment in terms of chemical composition, buffering capacity, competitive microbiota, and natural antagonist compounds (Naeem et al., 2012). The microbial populations of raw fruit commonly vary between 5 and 7 log CFU/g, where LAB constitute a small part (2–4 log CFU/g) of the autochthonous microbiota (Di Cagno et al., 2010a,b, 2011a,b).

Most probiotic bacteria are LAB, and among them, *Lactobacillus* is the most common genera (Argyri et al., 2013). According to FAO/WHO (2006), probiotics are non-pathogenic microorganisms, which exert a positive health benefit on the host when ingested in an adequate amount. The majority of the commercialized and most studied probiotics have been isolated from dairy products and human gastrointestinal tract (García-Ruiz et al., 2014). Although, dairy foods are recognized to be the best vehicle for the delivery of viable probiotics to the human gut, the increasing number of individuals with lactose intolerance, dyslipidemia, and vegetarianism reinforces the importance of the development of non-dairy probiotic products (Ranadheera et al., 2010; Peres et al., 2012), such as fruit juices. In fruit juices, the low pH (approx. 3.7) compared to the fairly neutral pH of milk (approx. 6.7) is possibly the chief determinant for the poor viability of probiotics in these matrices (Saarela et al., 2006). Raw fruit and their byproducts possess intrinsic physicochemical parameters that resemble those of the human gastrointestinal tract for some traits, such as the acidic environment and presence of anti-nutritional factors (tannins and phenols, Vitali et al., 2012). The natural adaptation to the intrinsic characteristics of fruit may help fruit-originating bacteria to survive during the processing and storage of fruit-based probiotic formulations as well as in the human stomach.

Various LAB have been isolated from fruit as follows: *Lactobacillus rossiae* from pineapple (Di Cagno et al., 2010a,b); *L. plantarum* from tomato, pineapple, plum, kiwi, papaya, grape, strawberry, and cherries (Di Cagno et al., 2008a,b, 2010a, 2011a,b; Naeem et al., 2012); *L. brevis* from tomato (Di Cagno et al., 2008b); and *Leuconostoc mesenteroides* subsp. *mesenteroides* and *Pediococcus pentosaceus* from cherries (Di Cagno et al., 2011b). The identification of LAB species in fruit is typically performed using molecular tools, particularly polymerase chain reaction (PCR)-based methods and 16S rRNA gene sequencing (Dusková et al., 2012). Matrix-assisted laser desorption/ionization–time of flight mass spectrometry (MALDI-TOF MS) has been recently introduced with marked success into routine clinical microbiological diagnosis of human pathogens (Bizzini et al., 2011; Welker, 2011; Nomura, 2015). However, studies reporting the application of MALDI-TOF MS for bacterial identification in food microbiology are still uncommon. The capability of this technique to identify bacteria isolated from food matrices not only at the genus and species level but also at the subspecies level reveals that MALDI-TOF MS could become a key tool in food microbiology and safety (Angelakis et al., 2011; Dusková et al., 2012).

This study aimed (i) to isolate and then identify LAB in fruit pulp processing byproducts using MALDI-TOF MS and 16S rRNA gene sequence analysis, as well as to verify the identification congruency between the two techniques; (ii) to assess the probiotic properties of selected *Lactobacillus* strains *in vitro*, including acid tolerance, bile tolerance, and capability to inhibit pathogenic food-related bacteria; and (iii) to verify the antibiotic resistance and growth behavior of the selected *Lactobacillus* strains in different cultivation media.

## Materials and methods

### Isolation of LAB

Samples (250 g) of fruit pulp processing byproducts of *Malpighia glabra* L. (barbados cherry), *Mangifera indica* L. (mango), *Annona muricata* L. (soursop), and *Fragaria vesca* L. (strawberry) were obtained from a company producing frozen fruit pulps located at the city of João Pessoa (Paraíba, Brazil). These byproducts were composed mostly of mashed peels and seeds as well as small amounts of mashed flesh. Initially, 25 g of each sample was suspended in 225 mL of sterile peptone water (0.1 g/100 mL) and homogenized using a stomacher (Model A440, Marconi Equip. Lab. Ltda., Piracicaba, Brazil) for 3 min at room temperature. Subsequently, serial dilutions (10^−2^–10^−5^) were performed using the same diluent, and 100 μL aliquots from each dilution were spread plated onto de Man, Rogosa, and Sharpe (MRS) agar (HiMedia, Mumbai, India) containing cysteine HCl (0.05 g/100 mL) and incubated anaerobically (Anaerobic System Anaerogen, Oxoid Ltda., Wade Road, UK) at 37°C for 48–72 h. At least five colonies presenting different morphologies were randomly isolated from MRS agar plates spread with the two highest serial dilutions of each type of fruit pulp byproduct. These isolates were maintained on MRS agar slants under refrigeration and further submitted to analysis of Gram staining, morphology, catalase production, and motility using standard procedures previously described (American Public Health (APHA), 2015). All of these analyses comprised a presumptive LAB identification step. All isolates presumptively identified as LAB (non-motile, catalase negative, Gram-positive cocci or rods) were stored at −20°C in MRS broth (HiMedia, Mumbai, India) containing glycerol (15 mL/100 mL) for further studies.

### Identification of LAB isolates

#### Identification using 16S rRNA gene sequence analysis

The bacterial genomic DNA was extracted using a Genomic DNA extraction kit (Promega Cooporation, Wisconsin, USA) according to the manufacturer's instructions. For the detection of 16S rRNA gene sequences, the following primers were used: 27F, 50-AGAGTTTGATCCTGGCTCAG-30, and 1492R, 50-GGTTACCTTGTTACGACTT-30. PCR was performed using a DNA thermocycler (Applied Biosystems, USA), and the reactions contained 0.5 μM of each primer, 0.2 mM dNTP mix, 1.5 mM MgCl_2_, and 1 U Taq DNA polymerase (Invitrogen, Germany) in a 50 μL final volume. The PCR was run under the following conditions: initial activation at 94°C for 2 min; denaturation step cycles at 94°C for 30 s; annealing step at 55°C for 1 min; extension step at 72°C for 1 min; and final cycle at 72°C for 10 min (Guo et al., 2010). The PCR products were purified using a DNA purification kit (Invitrogen, Germany) and sequenced using the 27F and 1492R primers in a sequencing reaction using the ABI Prism™ Bigdye™ terminator cycle sequencing reaction kit (Applied Biosystems, USA).

The resulting 1465 bp sequences were analyzed using the Pregap4 and Gap4 tools in the STADEN 1.6 software package and submitted to a search for similarity in the National Center for Biotechnology Information (NCBI) database using the blastn (nucleotide database) tool (Altschul et al., 1997; Guo et al., 2010) and Ribosomal Database Project (RDP). Bacterial identification was assumed when the query sequence showed similarity >97% for the 16S rRNA gene sequence (Gevers et al., 2005; Guo et al., 2010). Parcial 16S rRNA sequence was compared to known sequences in the NCBI Genbank database using the Local Alignment Search Tool (BLAST) algorithm (Altschul et al., 1990).

#### Identification using MALDI-TOF MS analysis

Initially, a standard protein extraction protocol adapted from Freiwald and Sauer (2009) was followed. Approximately 20 colonies of each LAB isolate culture were resuspended in 1.2 mL of 75% EtOH. After centrifugation (14,000 × g, 2 min, 4°C) and removal of the supernatant, proteins were extracted with 50 μL of an acetonitrile/formic acid/water mixture by vortexing for 1 min. The supernatant was then deposited in three wells of the sample plate at a volume of 1 μL and dried at room temperature, and the samples were then overlaid with 1 μL of a saturated alpha-cyano-4-hydroxycinnamic acid solution in acetonitrile:water:TFA (10 mg/mL; Bruker Daltonics, Germany).

The MALDI-TOF mass spectra measurements of samples were performed using a Bruker Biotyper 3.1 (Bruker Daltonics, Germany). External calibration of mass spectra was performed using *Escherichia coli* DH5 alpha standard peaks. Mass spectra were processed using MALDI Biotyper™ 3.1 software (Bruker Biotyper 3.1, Bruker Daltonics, Germany). The identification results were expressed by BioTyper log (scores) indicating the similarity of the unknown MALDI-TOF MS profile to available database entries. BioTyper logs (score) ≥2.3 and ≤3.0 indicate a highly probable identification at the species level; logs (score) ≥2.0 and ≤2.3 indicate secure genus identification, and probable species identification; logs (score) ≥1.7 and ≤2.0 imply probable genus identification; and logs (score) <1.7 imply no significant similarity between the unknown profile and any of the database entries. MALDI-TOF MS profile spectra for bacterial identification were automatelly compared to the BioTyper reference library of MALDI-TOF mass spectra by MALDI Biotype™ 3.1 software (Bruker Daltonics, Germany).

After the identification of LAB isolates, five different isolates with congruency of identification by the MALDI-TOF MS and 16S rRNA gene sequencing techniques that belonged to different species from the *Lactobacillus* genus, namely, *L. brevis, L. pentosus, L. paracasei, L. plantarum*, and *L. fermentum*, and that are commonly studied for probiotic properties, were selected for use in further assays of potential probiotic properties and growth behavior.

### Inoculum of lactobacilli and pathogenic bacteria

Initially, each *Lactobacillus* strain was grown anaerobically (Anaerobic System Anaerogen, Oxoid) in MRS broth at 37°C for 20–24 h (stationary growth phase), harvested through centrifugation (4500 *g*, 15 min, and 4°C), washed twice in sterile saline solution (0.85 g/100 mL) and resuspended in sterile saline solution to obtain cell suspensions with an OD reading at 660 nm (OD_660_) of 0.5. This suspension provided viable counts of approximately 8 log CFU/mL for each strain when pour plated in MRS agar.

The strains of the pathogenic bacteria *Staphylococcus aureus* (INCQS 00015, originally ATCC 25923), *Salmonella enterica* subsp. *enterica* serovar Typhimurium (INCQS 00150, originally ATCC 14028), *S. enterica* subsp. *enterica* serovar Enteritidis (INCQS 00258, originally 13076), *L. monocytogenes* (INCQS 00266, originally ATCC 7644), and *E. coli* (INCQS 00219, originally ATCC 8739) were obtained from the National Institute for Quality Control in Health (Oswaldo Cruz Foundation, Rio de Janeiro, Brazil). The stock cultures were maintained in Brain Heart Infusion (BHI) broth (HiMedia, Mumbai, India) containing glycerol (15 g/100 mL) at −20°C. Prior to use in antagonistic assays, each strain was aerobically grown in BHI broth at 37°C for 20–24 h, harvested through centrifugation (4500 *g*, 15 min, and 4°C), washed twice in sterile saline solution and resuspended in sterile saline solution to obtain cell suspensions with an OD_625_ of 0.1. This suspension provided viable cell counts of approximately 8 log CFU/mL for each strain when pour plated in BHI agar (HiMedia, Mumbai, India).

### Acid and bile salt tolerance assays

The tolerance to different pH values and bile salt concentrations was assessed by inoculating 1 mL aliquots of each tested *Lactobacillus* strain suspension in 10 mL of PBS (final viable cell counts of approximately 7 log CFU/mL) with pH adjusted to 2.0, 3.0 or 5.0 (using 1 M HCl) or supplemented with bile salts (Sigma-Aldrich Co., St. Louis, USA) at 1.0, 2.0 or 3.0% (w/v). The cells were incubated aerobically at 37°C under stirring (150 rpm). At different incubation periods (1, 2, and 3 h), 1 mL aliquots were removed from each system, serially diluted in sterile peptone water (10^−1^–10^−5^) and spread plated onto MRS agar for enumeration of viable cells. After an incubation period of 48 h at 37°C under anaerobiosis (Anaerobic System Anaerogen, Oxoid), the viable cells were counted, and the results were expressed as the log of the colony forming units per mL (log CFU/mL). For controls, *Lactobacillus* strains were cultivated in PBS at pH 7.2 (adjusted using 1 M HCl) and in MRS without bile salts (Jacobsen et al., 1999; Monteagudo-Mera et al., 2012).

### Antagonistic activity against pathogens

The antagonistic activity of the *Lactobacillus* strains against the indicator foodborne pathogenic bacteria was evaluated using the spot agar and well diffusion methods. For the spot agar test, a 2 μL-aliquot from each *Lactobacillus* strain suspension (approximately 7 log CFU/mL) cultivated overnight in MRS broth under anaerobiosis (Anaerobic System Anaerogen, Oxoid) was spotted on the surface of MRS agar containing 0.2% (w/v) glucose and 1.2% (w/v) agar and incubated anaerobically for 24 h at 37°C. A 1 mL-aliquot of each indicator bacterium suspension was then mixed with 18 mL of soft BHI agar (0.7% agar) (final viable count of approx. 5 log CFU/mL) and poured over the spot-inoculated MRS agar. The plates were incubated aerobically at 37°C for 48 h. The antagonistic activity was recorded as the diameter (mm) of growth inhibition zones around each spot (Jacobsen et al., 1999). Uninoculated MRS agar was used as a negative control.

For the well diffusion method, *Lactobacillus* strains were first cultivated for 18 h in MRS broth under anaerobiosis (Anaerobic System Anaerogen, Oxoid), and the supernatants of these cultures were collected by centrifugation (15,000 g, 15 min, and 4°C). A 1 mL aliquot of each indicator bacterium suspension was then incorporated into 20 mL BHI soft agar plates (final viable counts of approx. 5 log CFU/mL), and 50 μL aliquots of lactobacilli supernatants were dispensed into wells (5 mm diameter and 5 mm depth; drilled using sterile glass cannulas) in BHI agar. The plates were aerobically incubated at 37°C for 48 h. After the incubation period, the antagonistic activity was recorded as the diameter (mm) of growth inhibition zones around each well. In this assay, MRS broth was used as the negative control (Vitali et al., 2012).

In both, the spot agar and well diffusion assays, a free growth inhibition zone with a diameter greater than 1 mm (around the spot or well) was considered as positive inhibitory activity (Jacobsen et al., 1999). All the five tested lactobacilli isolates were tested for the capability to inhibit each other.

### Antibiotic susceptibility testing

The minimum inhibitory concentrations of ampicillin, chloramphenicol, clindamycin, erythromycin, gentamycin, kanamycin, streptomycin, and tetracycline (European Food Safety Authority (EFSA), 2012) against the selected lactobacilli strains were determined using a broth microdilution test previously described (CLSI, 2012) with a minor modification related to the growth media and incubation atmosphere. Approximately 50 μL of each antibiotic solution was dispensed into each well of a 96-well microplate containing 100 μL of MRS broth. Subsequently, a 50 μL-culture aliquot of each test lactobacilli isolate was added to each well (final viable cell count of approximately 7 log CFU/mL). The microplate was loosely wrapped with cling wrap to prevent bacterial dehydration. Each plate included a control (without antibiotic), an inoculated sample (positive control) or an uninoculated sample (negative control). The system was anaerobically (Anaerobic System Anaerogen, Oxoid) and statically incubated at 37°C for 48 h. Subsequently, the bacterial growth was visually observed, and the MIC of each antibiotic was confirmed as its lowest concentration capable of inhibiting visible bacterial growth. The MIC cut-off values of European Food Safety Authority (EFSA) (2012) were considered to categorize the lactobacilli strains as susceptible or resistant to each tested antibiotic. Each isolate was defined as susceptible when it was inhibited at a concentration (μg/mL) of a specific antibiotic equal to or lower than the established cut-off value, and each isolate was defined as resistant when it was inhibited at a concentration (μg/mL) of a specific antibiotic higher than the established cut-off value (European Food Safety Authority (EFSA), 2012).

### Growth kinetics in MRS broth and general edible medium broth

The growth kinetics of *Lactobacillus* strains were assessed in MRS broth and in general edible medium (GEM) broth (40 g/L glucose, 30 g/L soya peptone, 7 g/L yeast extract, and 1 g/L MgSO_4_.7H_2_O in 0.01 mol/L K-phosphate buffer; pH 6.3 ± 0.2; Saarela et al., 2004) using two different volume scales, i.e., 200 and 2000 mL. Aliquots of each lactobacilli strain suspension were inoculated (1% v/v; final viable cell count of approximately 7 log CFU/mL) in MRS or GEM broth and incubated aerobically at 37°C under stirring (150 rpm) for 48 h. At different incubation time intervals (16, 24, and 48 h), samples were taken (1 mL) and serially diluted (10^−1^–10^−5^) in sterile peptone (0.1 g/100 mL), spread plated onto MRS agar, and incubated anaerobically (Anaerobic System Anaerogen, Oxoid) at 37°C for 48 h. After the incubation period, the viable cells were counted, and the results were expressed as the log CFU/mL.

### Reproducibility and statistical analysis

All assays were performed in triplicate in two independent experiments (repetitions), and the results are expressed as the average of the tests. Statistical analyses were performed to determine significant differences (*P* ≤ 0.05) among obtained results using the Student's *t*-test or ANOVA followed by Tukey's *post hoc* test. These analyses were performed using Graphpad Prism 6.0 software.

## Results

### Identification of LAB

A total of 50 isolates of LAB, comprising 10 isolates from each type of pulp fruit processing byproduct source, were randomly selected for identification (Table 1 and Table S1). Species belonging to *Lactobacillus* genus were the most predominant (41/50 isolates; 82%) identified LAB. In pineapple and barbados cherry pulp byproducts only a few isolates were identified as *Lactococcus lactis* (one and two isolates, respectively) and in the mango pulp byproduct, one isolate was identified as *P. pentosaceus*, and two isolates were identified as *L. mesenteroides*. The following *Lactobacillus* species were found for each type of fruit pulp byproduct: *L. fermentum* and *L. nagelii* in pineapple pulp byproduct; *L. plantarum, L. brevis, L. fermentum*, and *L. nagelii* in barbados cherry pulp byproduct; *L. fermentum, L. casei, L. paracasei*, and *L. nagelii* in soursop pulp byproduct; *L. plantarum, L. pentosus*, and *L. nagelii* in mango pulp byproduct; and *L. fermentum* in strawberry pulp byproduct. Considering the total number of isolates identified as belonging to *Lactobacillus* genus, the following rank for frequency of species identification was observed: *L. fermentum* > *L. plantarum*/*L. nagelii* > *L. brevis* > *L. pentosus*/*L. paracasei*/*L. casei*.

**Table 1:** **Identification of lactic acid bacteria isolates from different fruit pulp processing byproducts (total number of isolates from each source, technique applied for identification and number of isolates identified by each applied identification technique)**.

**By-product source**	**Total number of isolates**	**Identification technique**			
		**MALDI-TOF**	**Number of identified isolates**	**16S gene**	**Number of identified isolates**
Pineapple	10	*L. fermentum*	6	*L. fermentum*	9
		*L. lactis*	1	*L. lactis*	1
		*L. nagelii*	1	–	0
		*Lactobacillus* spp.	2	–	0
Barbados cherry	10	*L. plantarum*	3	*L. plantarum*	3
		*L. fermentum*	2	*L. fermentum*	2
		*L. brevis*	2	*L. brevis*	2
		*L. nagelii*	1	*L. nagelii*	1
		*L. lactis*	2	*L. lactis*	2
Soursop	10	*Lactobacillus* spp.	2	*Lactobacillus* spp.	0
		*L. paracasei*	2	*L. paracasei*	1
		*L. nagelii*	4	*L. nagelii*	4
		*L. fermentum*	2	*L. fermentum*	2
		–	-	*L. casei*	1
Mango	10	*L. pentosus*	1	*L. pentosus*	1
		*L. mesenteroides*	3	*L. mesenteroides*	3
		*P. pentosaceus*	1	*P. pentosaceus*	1
		*L. fermentum*	1	*L. fermentum*	1
		*L. nagelii*	1	*L. nagelii*	1
		*L. plantarum*	3	*L. plantarum*	3
Strawberry	10	*L. fermentum*	7	*L. fermentum*	10
		*Lactobacillus* spp.	3	–	0

(–), genera/species that was not identified by the technique.

The average congruency between MALDI-TOF MS and 16S rRNA gene sequence analysis in the identification of LAB species reached 86.1% (Table 2). Isolates of *L. plantarum, L. brevis, L. pentosus, L. lactis*, and *L. mesenteroides* were identified with 100% congruency. The lowest congruency (50%) was obtained for *L. casei* and *L. paracasei*. The low identification congruency (75%) for *L. fermentum* was due to the inability of MALDI-TOF MS to identify this species beyond the genus level (*Lactobacillus* spp.). All isolates that were not identified to the species level by MALDI-TOF MS were further identified as *L. fermentum* by 16S rRNA gene sequence analysis. In this study, there was an 86 and 100% efficiency of LAB species identification by the MALDI-TOF MS and 16S rRNA gene analysis techniques, respectively. Without considering the *L. casei* and *L. paracasei* isolates, which were distinctly identified in MALDI-TOF MS and 16S-rRNA gene analysis, both techniques showed similar 100% efficiency in LAB species identification.

**Table 2:** **Number of identified lactic acid species from fruit pulp processing byproducts according to each applied identification technique and congruency of identification between the applied identification techniques**.

**Genera/species of lactic acid bacteria**	**Number of strains identified by MALDI-TOF**	**Number of strains identified by 16S gene**	**%Congruency**
*L. fermentum*	18	24	75
*L. plantarum*	6	6	100
*L. brevis*	2	2	100
*L. nagelii*	7	6	86
*L. paracasei*	2	1	50
*L. casei*	0	1	50
*L. pentosus*	1	1	100
*L. lactis*	3	3	100
*L. mesenteroides*	3	3	100
*P. pentosaceus*	1	1	100

To perform the second stage of this study, five strains identified as different *Lactobacillus* species, namely, *L. brevis* 59, *L. pentosus* 129, *L. paracasei* 108, *L. plantarum* 49, and *L. fermentum* 111, were selected for inclusion in further assays.

### Tolerance to acidic conditions and bile salt concentrations

The testing of the tolerance of the *Lactobacillus* strains to different pH values revealed sharp decreases in viable counts (low survival rate) during the 3 h assessed incubation period. At pH 3.0, the tested *Lactobacillus* strains presented variable declines in viable counts over time. In this condition, *L. brevis* 59 presented the highest (*P* ≤ 0.05) viable counts during the 3 h assessed incubation period (good tolerance), while *L. pentosus* 129, *L. paracasei* 108, and *L. plantarum* 111 displayed sharp decreases in viable counts after the 2 or 3 h incubation. At pH 5.0, the five tested strains survived during the 3 h incubation (good tolerance) (Table 3). In most cases, the strains displayed similar (*P* > 0.05) viable counts when cultivated in both pH 5.0 and 7.2.

Table 3**Viable cell counts (*n* = 3, mean values ± standard deviation; log CFU/g) of different lactobacilli strains from pulp fruit processing byproducts when challenged with different pH values and bile salt concentrations for different exposure time periods and antagonistic activities, as expressed in diameter (mm) of growth inhibition zones (± standard deviation), toward pathogenic bacteria as measured by spot agar and well diffusion assays**.

**Table:** 

**Strains**	**Control (pH 7.2)**			**pH 2.0**			**pH 3.0**			**pH 5.0**		
	**Exposure time (h)**			**Exposure time (h)**			**Exposure time (h)**			**Exposure time (h)**		
	**1**	**2**	**3**	**1**	**2**	**3**	**1**	**2**	**3**	**1**	**2**	**3**
**pH VALUES**												
*L. brevis* 59	7.5 (±0.2)^A^^b^	7.5 (±0.3)^A^^c^	7.4 (±0.3)^A^^c^	<2 (±0.0)^A^^a^	<2 (±0.0)^A^^a^	<2 (±0.0)^A^^a^	7.5 (±0.2)^B^^d^	7.2 (±0.1)^B^^c^	6.4 (±0.4)^A^^c^	7.4 (±0.2)^B^^b^	7.2 (±0.3)^B^^c^	6.1 (±0.1)^A^^b^
*L. pentosus* 129	6.9 (±0.3)^A^^a^	6.5 (±0.3)^A^^a^	6.7 (±0.2)^B^^a^	<2 (±0.0)^A^^a^	<2 (±0.0)^A^^a^	<2 (±0.0)^A^^a^	6.1 (±0.2)^C^^a^	4.1 (±0.7)^B^^a^	<2 (±0.0)^A^^a^	6.5 (±0.7)^C^^a^	5.8 (±0.2)^A^^a^	5.7 (±0.0)^A^^a^
*L. paracasei* 108	7.1 (±0.3)^A^^b^	7.3 (±0.2)^A^^c^	7.2 (±0.2)^A^^bc^	3.7 (±0.1)^B^^b^	<2 (±0.0)^A^^a^	<2 (±0.0)^A^^a^	3.7 (±0.2)^B^^a^	<2 (±0.0)^A^^a^	<2 (±0.0)^A^^a^	7.3 (±0.3)^A^^b^	7.1 (±0.2)^A^^c^	6.9 (±0.2)^A^^c^
*L. plantarum* 49	6.8 (±0.1)^A^^b^	6.6 (±0.2)^A^^b^	6.7 (±0.1)^A^^b^	6.7 (±0.1)^B^^d^	<2 (±0.0)^A^^a^	<2 (±0.0)^A^^a^	6.7 (±0.1)^B^^c^	<2 (±0.0)^A^^a^	<2 (±0.0)^A^^a^	7.2 (±0.1)^B^^b^	6.5 (±0.2)^A^^b^	6.0 (±0.2)^A^^b^
*L. fermentum* 111	6.8 (±0.1)^A^^a^	6.4 (±0.2)^A^^a^	6.3 (±0.2)^A^^a^	6.1 (±0.2)^B^^c^	<2 (±0.0)^A^^a^	<2 (±0.0)^A^^a^	6.3 (±0.3)^A^^b^	6.3 (±0.0)^A^^b^	6.4 (±0.3)^A^^b^	6.7 (±0.3)^A^^a^	6.3 (±0.3)^A^^b^	6.1 (±0.2)^A^^d^
**Strains**	**Control (0.00%)**			**0.15%**			**0.30%**			**1.00%**		
	**Exposure time (h)**			**Exposure time (h)**			**Exposure time (h)**			**Exposure time (h)**		
	**1**	**2**	**3**	**1**	**2**	**3**	**1**	**2**	**3**	**1**	**2**	**3**
**BILE SALTS (CONCENTRATION—%)**												
*L. brevis* 59	7.2 (±0.1)^A^^d^	6.8 (±0.2)^A^^b^	7.1 (±0.2)^A^^b^	7.2 (±0.3)^A^^c^	7.1 (±0.3)^A^^b^	6.8 (±0.1)^A^^b^	7.0 (±0.3)^A^^b^	7.2 (±0.1)^A^^b^	7.2 (±0.2)^A^^b^	7.6 (±0.1)^A^^b^	7.3 (±0.1)^A^^a^	7.4 (±0.2)^A^^b^
*L. pentosus* 129	7.1 (±0.1)^A^^c^	7.2 (±0.2)^A^^b^	7.5 (±0.3)^A^^b^	7.1 (±0.2)^A^^c^	7.0 (±0.3)^A^^b^	6.9 (±0.2)^A^^b^	7.1 (±0.1)^A^^b^	7.1 (±0.2)^A^^b^	6.9 (±0.3)^A^^b^	7.1 (±0.1)^A^^a^	7.2 (±0.2)^A^^a^	7.0 (±0.2)^A^^a^
*L. paracasei* 108	6.8 (±0.1)^A^^a^	7.1 (±0.1)^B^^a^	7.3 (±0.2)^C^^b^	6.8 (±0.2)^A^^b^	6.4 (±0.0)^A^^a^	6.2 (±0.2)^A^^b^	6.8 (±0.4)^A^^a^	7.0 (±0.3)^A^^b^	7.2 (±0.2)^A^^b^	7.0 (±0.1)^A^^a^	6.9 (±0.2)^A^^a^	7.1 (±0.1)^A^^a^
*L. plantarum* 49	7.1 (±0.1)^A^^c^	7.0 (±0.1)^A^^b^	7.3 (±0.1)^A^^a^	7.0 (±0.1)^A^^c^	7.2 (±0.1)^A^^b^	7.1 (±0.2)^A^^c^	7.1 (±0.2)^A^^b^	7.0 (±0.1)^A^^a^	7.1 (±0.1)^A^^b^	7.1 (±0.2)^A^^a^	7.2 (±0.3)^A^^a^	6.8 (±0.3)^A^^a^
*L. fermentum* 111	6.9 (±0.2)^A^^b^	7.2 (±0.3)^A^^a^	7.4 (±0.3)^A^^a^	6.8 (±0.3)^A^^a^	6.7 (±0.3)^A^^a^	6.9 (±0.3)^B^^a^	7.2 (±0.1)^A^^b^	6.9 (±0.1)^A^^b^	6.8 (±0.3)^A^^a^	6.8 (±0.2)^A^^a^	6.9 (±0.1)^A^^a^	6.7 (±0.2)^A^^a^

**Table:** 

**Inhibitory strain**	**Indicator Strain**									
	***S. aureus* INCQS 00015**		***S. typhi* INCQS 00150**		***S. enteritidis* INCQS 00258**		***L. monocytogenes* INCQS 00266**		***E. coli* INCQS 00219**	
	**Spot agar**	**Well diffusion**	**Spot agar**	**Well diffusion**	**Spot agar**	**Well diffusion**	**Spot agar**	**Well diffusion**	**Spot agar**	**Well diffusion**
**ANTAGONISTIC ACTIVITY**										
*L. brevis* 59	4.0 (±1.0)^a^	<1 (±0.0)^a^	6.0 (±0.7)^a^	2.0 (±0.3)^a^	5.0 (±1.0)^a^	1.5 (±0.3)^a^	4.0 (±0.5)^a^	2.5 (±0.3)^a^	4.5 (±1.0)^a^	2.0 (±0.3)^a^
*L. pentosus* 129	7.0 (±0.9)^d^	4.0 (±0.5)^c^	8.5 (±0.5)^c^	2.5 (±0.4)^a^^b^^c^	10.5 (±1.0)^c^	3.0 (±0.5)^b^	8.0 (±1.0)^c^	4.0 (±0.5)^b^	6.5 (±0.8)^b^	4.5 (±0.5)^c^
*L. paracasei* 108	7.0 (±1.0)^d^	<1 (±0.0)^a^	8.0 (±0.4)^b^^c^	3.0 (±0.5)^b^^c^	8.3 (±0.6)^b^	3.0 (±0.5)^b^	8.0 (±0.7)^c^	2.0 (±0.5)^a^	8.5 (±1.0)^c^	4.0 (±0.7)^c^
*L. plantarum* 49	9.0 (±0.5)^c^	2.8 (±0.5)^b^	7.5 (±0.5)^b^	2.5 (±0.3)^a^^b^	10.8 (±1.0)^c^	3.5 (±0.3)^b^	8.2 (±0.8)^c^	3.0 (±0.8)^a^^b^	5.3 (±0.6)^a^	3.0 (±0.5)^b^
*L. fermentum* 111	5.0 (± 1.0)^b^	<1 (±0.0)^a^	6.7 (±0.6)^a^^b^	2.0 (±0.5)^a^	8.5 (±0.5)^b^	3.0 (±0.6)^b^	6.0 (±1.0)^b^	2.0 (±0.5)^a^	6.0 (±0.5)^a^^b^	1.5 (±0.3)^a^

A−B *: different superscript capital letters in the same row denote differences (P ≤ 0.05) in counts obtained in each assayed stress condition (pH value or bile salt concentration) for each tested lactic acid bacteria based on Tukey's test*;

a−d : different superscript small letters in the same column denote differences (P ≤ 0.05) for the different tested lactic acid bacteria based on Tukey's test.

Overall, the exposure to the different bile salt concentrations did not result in decreases (*P* > 0.05) in the initial viable counts of the tested *Lactobacillus* strains during the 3 h incubation (good tolerance), and no differences (*P* > 0.05) were found compared to the systems not exposed to bile salts (Table 3).

### Antagonistic activity against pathogenic bacteria

The five tested *Lactobacillus* strains presented inhibitory activities against all the selected target pathogenic bacteria strains in spot agar assay, and the inhibitory effects (diameter of growth inhibition zones) varied among inhibitors and indicator strains. In the spot agar assay, the growth inhibition zone diameters displayed by the five tested *Lactobacillus* strains were ≥4 mm against all target pathogenic bacteria and reached ≥8.0 mm in most cases. Similarly, the cell-free supernatants of all *Lactobacillus* strains presented inhibitory activity against the target bacteria in the well diffusion assay with growth inhibition zone diameters varying from 1.5 to 4.5 mm. The only exceptions were the cell-free supernatants of *L. brevis* 59 and *L. fermentum* 111, which did not inhibit the growth of *S. aureus* INCQS 00015 (Table 3). The diameters of the growth inhibition zones were always greater in spot agar assays than in well diffusion assays.

The strongest antagonistic activities in spot agar and well diffusion assays were displayed by *L. pentosus* 129 and *L. plantarum* 49. Considering the average diameter of growth inhibition zones detected by the spot agar and well diffusion assays, the following rank of sensitivity among the target foodborne pathogenic bacteria was observed: *Salmonella* Enteritidis > *Salmonella* Typhimurium > *L. monocytogenes* > *E. coli* > *S. aureus*.

### Antibiotic resistance

The studied lactobacilli strains did not show resistance to ampicillin, chloramphenicol, or streptomycin, except for *L. paracasei* 108 that presented resistance to ampicillin. Three out of the five strains were resistant to gentamycin (*L. plantarum* 49, *L. paracasei* 108, and *L. pentosus* 129) and tetracycline (*L. brevis* 59, *L. paracasei* 108, and *L. fermentum* 111), and two strains were resistant to clindamycin (*L. brevis* 59 and *L. paracasei* 108). All of the strains were resistant to kanamycin and erythromycin (Table 4). Overall, the resistance profiles to antibiotics varied among the lactobacilli strains.

**Table 4:** **MIC of different antibiotics against different lactobacilli strains from pulp fruit processing byproducts**.

**Strains**	**Antibiotics**							
	**Ampicillin**	**Chloramphenicol**	**Clindamycin**	**Erythromycin**	**Gentamycin**	**Kanamycin**	**Streptomycin**	**Tetracycline**
*L. brevis* 59	2^S^	2^S^	32^R^	512^R^	16^S^	256^R^	16^S^	512 ^R^
*L. pentosus* 129	2^S^	2^S^	1^S^	512^R^	64^R^	128^R^	64^S^	256^S^
*L. paracasei* 108	64^R^	2^S^	64^R^	512^R^	64^R^	256^R^	64^S^	512 ^R^
*L. plantarum* 49	2^S^	2^S^	1^S^	512^R^	64^R^	128^R^	64^S^	512^S^
*L. fermentum* 111	1^S^	2^S^	1^S^	256^R^	8^S^	256^R^	16^S^	512 ^R^

Resistant^(R)^ or Sensitive^(S)^ profile according to the cut-offs recommended by European Food Safety Authority (EFSA) (2012).

### Growth in MRS and GEM broth

The viable counts of *Lactobacillus* strains in MRS and GEM broth at two different medium volume scales (200 and 2000 mL) during 48 h are shown in Table 5. In most cases, the viable counts were greater (*P* ≤ 0.05) in MRS broth than in GEM broth during the assessed incubation period. However, the viable counts of the five strains in MRS and GEM broths were maintained at ≥6.7 log CFU/mL (6.7–7.2 CFU/mL) during the monitored incubation period, and the viable counts were close (*P* > 0.05) to those observed at the beginning of the experiments (approx. 7 log CFU/mL). Overall, at the two different assessed MRS and GEM broth volumes, the *Lactobacillus* strains presented similar viable counts (*P* > 0.05) during the 48 h cultivation period with increasing counts up to 16 h of cultivation followed by a decline at the later assessed cultivation periods.

**Table 5:** **Viable cell counts (*n* = 3, mean values ± standard deviation; log CFU/g) of lactobacilli isolates from pulp fruit byproducts when cultivated in de Man, Rogosa, and Sharpe (MRS) and in general edible medium (GEM) broths at low-scale (200 mL) and medium-scale (2000 mL) volumes at 37°C during 48 h (initial viable cell counts of approx. 7 log CFU/mL)**.

**Strain**	**Time of cultivation (h)**	**Cultivation media (volume)**			
		**MRS (200 mL)**	**GEM (200 mL)**	**MRS (2000 mL)**	**GEM (2000 mL)**
*L. plantarum* 149	16	8.5 (±0.3)^B^^b^	7.8 (±0.2)^B^^a^	8.1 (±0.2)^B^^a^	8.2 (±0.2)^B^^a^
	24	8.3 (±0.2)^B^^b^^*^	7.7 (±0.3)^B^^a^	7.4 (±0.3)^A^^a^^*^	7.8 (±0.1)^A^^a^
	48	7.6 (±0.3)^A^^b^	6.9 (±0.3)^A^^a^^*^	7.1 (±0.2)^A^^a^	7.5 (±0.2)^A^^a^^*^
*L. brevis* 59	16	8.2 (±0.3)^B^^b^	7.5 (±0.3)^A^^a^	8.5 (±0.1)^B^^b^	7.9 (±0.1)^B^^a^
	24	8.6 (±0.2)^B^^b^	7.6 (±0.2)^A^^a^	8.3 (±0.2)^B^^b^	7.4 (±0.3)^A^^a^
	48	7.4 (±0.3)^A^^b^	6.5 (±0.3)^Ba^^*^	7.2 (±0.3)^A^^a^	7.3 (±0.2)^A^^a^^*^
*L. paracasei* 108	16	7.9 (±0.2)^B^^a^	7.7 (±0.2)^B^^a^	8.6 (±0.1)^C^^b^	7.8 (±0.1)^C^^a^
	24	8.4 (±0.3)^B^^b^	7.6 (±0.2)^B^^a^	8.1 (±0.1)^B^^b^	7.4 (±0.2)^B^^a^
	48	7.3 (±0.3)^A^^a^	7.1 (±0.3)^A^^a^	7.4 (±0.4)^A^^b^	6.8 (±0.2)^A^^a^
*L. fermentum* 111	16	8.4 (±0.2)^B^^a^	8.3 (±0.1)^B^^a^	8.0 (±0.1)^B^^a^	8.4 (±0.3)^C^^a^
	24	8.1 (±0.3)^Aba^^*^	8.0 (±0.2)^B^^a^	7.4 (±0.3)^A^^a^^*^	8.1 (±0.2)^B^^b^
	48	7.6 (±0.3)^A^^a^	7.2 (±0.2)^A^^a^	6.8 (±0.3)^A^^a^	7.6 (±0.2)^A^^b^
*L. pentosus* 129	16	8.2 (±0.3)^A^^b^	7.7 (±0.2)^B^^a^	8.2 (±0.1)^B^^b^	7.9 (±0.2)^B^^a^
	24	8.6 (±0.2)^A^^b^^*^	7.4 (±0.3)^B^^a^	7.8 (±0.2)^A^^a^^*^	7.4 (±0.3)^A^^B^^a^
	48	7.4 (±0.3)^A^^b^^*^	6.8 (±0.3)^A^^a^	7.7 (±0.3)^A^^b^^*^	6.9 (±0.3)^A^^a^

A−C *: different superscript capital letters in the same row denote differences (P ≤ 0.05) in counts obtained in different cultivation media and volume or in the same cultivation media but with different volumes based on Student's t-test*;

a−b *: different superscript small letters in the same column for the same cultivation media and volume denote differences (P ≤ 0.05) in counts obtained for each lactic acid bacteria based on Student's t test*;

* *: denotes differences (P ≤ 0.05) in counts of the same lactic acid bacteria cultivated in the same broth but in low- and medium-scale volumes based on Student's t-test*.

## Discussion

Recently, MALDI-TOF MS has been introduced for the identification of microorganisms, and it has been suggested as a key tool in food safety and control (Jadhav et al., 2015; Nomura, 2015). However, studies showing the performance and reproducibility of this method to identify LAB species from food sources are still scarce. It has been reported that a polyphasic strategy based on molecular techniques is necessary for accurate species designation within the LAB group (Singh et al., 2009). Thus, 16S RNA gene sequence analysis has become a routine tool in LAB species identification (Dusková et al., 2012). In the present study, the results showed 100% congruency between MALDI-TOF MS and 16S rRNA gene analysis in identification of *L. plantarum, L. brevis, L. pentosus, L. lactis*, and *L. mesenteroides*, but divergence was found between the two methods for the identification of *L. casei, L. paracasei*, and *L. fermentum*. The difficulty in the identification of *L. casei*/*paracasei* by MALDI-TOF MS or 16S rRNA gene sequence analysis and cross reactions for these species in PCR-based methods have already been reported (Sisto et al., 2009; Dusková et al., 2012). Angelakis et al. (2011) found discrepancy in identification of *L. paracasei* instead of *L. casei* by MALDI-TOF MS, and this discordance was explained to be probably related with the number of species (*L. casei, L. paracasei, L. rhamnosus, L. zeae*) forming the *L. casei* group, which cannot be distinguished by conventional phenotypic properties (Klein et al., 1998; Holzapfel et al., 2001). Taxonomic controversies on to reject (Dicks et al., 1996) or to retain (Dellaglio et al., 2002) the species name *L. paracasei* may also difficult the denomination of a same strain. These difficulties in correct identification of lactobacilli have led to difficulties in classification of *Lactobacillus* strains (Schillinger et al., 2003).

Considering the view of the controversy on the nomenclature and taxonomy of *L. casei*-related taxa, it is possible that isolates assigned to *L. casei* should actually be classified as *L. paracasei* (Sisto et al., 2009; Dusková et al., 2012). Another interesting finding in the present study was the genus-level identification (*Lactobacillus* spp.) by MALDI-TOF MS of seven isolates, which were further assigned by 16S rRNA gene sequence analysis as *L. fermentum*. An earlier study observed similar results and reported a MALDI-TOF MS log (score) indicating probable genus identification for one *Lactobacillus* strain, which was further assigned by PCR as *L. fermentum* (Dusková et al., 2012). Thus, the discriminatory inability of MALDI-TOF MS to identify species of some *Lactobacillus* strains, which were further identified as *L. fermentum* by 16S rRNA gene analysis, suggests that clarification of MALDI-TOF MS outputs using well-known molecular techniques is still required.

The observed prevalence of *Lactobacillus* species in the autochthonous LAB microbiota of fruit pulp processing byproducts has already been reported for raw fruit and vegetables (Vitali et al., 2012; Argyri et al., 2013). Although, the epiphytic microbial population of plants is largely subjected to fluctuations of physicochemical and nutritional conditions, each fruit and vegetable harbors a dominant and constant microbiota (Yang et al., 2000; Dusková et al., 2012). Supporting our data, *L. plantarum, L. brevis, L. fermentum*, and *L. paracasei* are cited among the most frequent lactobacilli isolated from fruit and vegetables (Vitali et al., 2012; Argyri et al., 2013).

*In vitro* studies of tolerance to harsh acidic conditions and bile contents normally found in the stomach and upper parts of the intestine, respectively, together with the capability to inhibit pathogenic bacteria, have been successfully used to select potentially probiotic LAB (Mättö et al., 2006; Tuo et al., 2013). In the present study, none of the five selected *Lactobacillus* strains (*L. brevis* 59, *L. pentosus* 129, *L. paracasei* 108, *L. plantarum* 49, and *L. fermentum* 111) survived at pH 2.0 after 2 h of exposure. The tolerance assays at pH 3.0 revealed a clear separation of a group composed of three strains (*L. brevis* 59, *L. pentosus* 129, and *L. fermentum* 111) that presented good tolerance and another group of two strains (*L. paracasei* 108 and *L. plantarum* 49) that showed a sharp loss of cell viability after 2 h of exposure. In agreement with these results, some studies have verified that the capability of different *Lactobacillus* strains to maintain cell viability varies greatly between pH 2.0 and 3.0 (Jacobsen et al., 1999; Monteagudo-Mera et al., 2012). In the stomach, probiotic strains do not necessarily encounter as low pH as 2.0 because the environment can be buffered by various food components, increasing the gastric pH (Zarate et al., 2000). Components of gastric juice may also confer some protective effect on bacterial cell viability (Conway et al., 1987). In this sense, the survival of probiotic strains during gastric transit depends on both their intrinsic tolerance to the hostile conditions found therein and the food matrix harboring them.

The studied *Lactobacillus* strains presented good tolerance to 0.15, 0.30, and 1.00% bile salt concentrations with no delay in growth. Bile tolerance is an important characteristic in *Lactobacillus* species enabling them to survive, metabolize and grow during the gastrointestinal transit as well as to exert their beneficial effects on the host (Charteris et al., 1998; Argyri et al., 2013). Interestingly, some studies have found a relationship between high bile salt tolerance and the capability to hydrolyze bile salts in *Lactobacillus* strains (Sridev et al., 2009; Argyri et al., 2013), and this later property has been correlated to cholesterol lowering effects (Begley et al., 2006).

The five tested *Lactobacillus* strains displayed the capability to inhibit pathogenic bacteria, including *E. coli, L. monocytogenes, Salmonella* Enteritidis, *Salmonella* Typhimurium, and *S. aureus*, in the spot agar and/or well diffusion assays, but the nature of the inhibitory substance(s) remains unknown. The capability to produce antimicrobial compounds, such as organic acids, short chain fatty acids and bacteriocins, is one of the functional properties used to characterize probiotics (Argyri et al., 2013). The production of some metabolites with antimicrobial properties by probiotic bacteria can be beneficial for food preservation and the prevention of the growth of foodborne pathogens (Monteagudo-Mera et al., 2012).

Among the required properties by which specific strains can be considered as potential probiotics is that they do not harbor acquired and transferable (added genes) antibiotic resistances (European Food Safety Authority (EFSA), 2012; Gueimonde et al., 2013). The resistance of all five strains to erythromycin and kanamycin, as well as the resistance of two strains to clindamycin, are of concern. Resistance to tetracycline or gentamycin was also observed for three strains. The nature of the resistance warrants further studies before any of the resistant strains can be considered safe for human use.

Studies of the growth behavior of the selected *Lactobacillus* strains revealed a repeatedly similar growth pattern in both MRS and GEM broths. The similar growth behavior of the strains in MRS broth, a well-known expensive laboratory medium for *Lactobacillus* cultivation, and in GEM broth, an inexpensive media containing only food-grade ingredients (Saarela et al., 2006; Pimentel et al., 2012), is noteworthy. Particularly, the verified growth behavior in GEM broth encourages further studies for scale-up and optimization of the production of highly concentrated forms of the tested *Lactobacillus* strains in this medium for possible direct vat applications (as highly concentrated frozen cultures or as freeze-dried cultures) in formulations.

In conclusion, the results of this study showed that the LAB forming the microbiota of the studied fruit pulp processing byproducts mostly belonged to the *Lactobacillus* genus and included the *L. fermentum, L. plantarum, L. nagelii, L. brevis, L. pentosus, L. paracasei*, and *L. casei* species. MALDI-TOF MS and 16S rRNA gene sequence analysis revealed high congruency of LAB species identification. Overall, the selected *Lactobacillus* strains revealed the following desirable probiotic-related properties: tolerance to different acidic conditions and bile salt concentrations; good growth in both laboratory and edible growth media; and capacity to inhibit distinct pathogenic bacteria despite their variable susceptibility to different antibiotics. Thus, these data suggested that most of the assessed *Lactobacillus* strains are good candidates for further studies including fermentation and technological characteristics, survival in different food matrices and impacts on quality characteristics, as well as for *in vivo* studies to verify their potential health benefits. Finally, fruit pulp processing byproducts may be considered potential sources of *Lactobacillus* strains possessing interesting probiotic-related properties.

## Author contributions

Conceived and designed the experiments: ED, MS. Performed the experiments: EG, WL, DX, WD, KD, MD, BL, RP, OF. Analyzed the data: ED, BL, EG, MM, MS. Drafted the paper: ED, EG, MM, MS.

### Conflict of interest statement

The authors declare that the research was conducted in the absence of any commercial or financial relationships that could be construed as a potential conflict of interest.
