# Supplementary material for: Identification of Lactic Acid Bacteria in Fruit Pulp Processing Byproducts and Potential Probiotic Properties of Selected Lactobacillus Strains
Source: Front Microbiol. 2016 Aug 30;7:1371. doi: 10.3389/fmicb.2016.01371 (PMC5003889; doi:10.3389/fmicb.2016.01371)
Supplement: Supplementary file 1 [file Table1.pdf]

Table S1. Identification of lactic acid bacteria from fruit pulp processing byproducts (source, identification technique and congruency between applied techniques of identification).

Byproduct source	Strain code	Identification technique		Congruency
		**MALDI-TOF**	**16S-rRNA**	
Pineapple	40	*L. fermentum*	*L. fermentum*	yes
	52	*L. fermentum*	*L. fermentum*	yes
	56	*L. fermentum*	*L fermentum*	yes
	220	*L. fermentum*	*L. fermentum*	yes
	250	*L. fermentum*	*L. fermentum*	yes
	255	*Lactobacillus* spp.	*L. fermentum*	yes
	256	*Lactobacillus* spp.	*L. fermentum*	yes
	263	*L. fermentum*	*L. fermentum*	yes
	273	*L. nagelii*	*L. fermentum*	no
	278	*L. lactis*	*L. lactis*	yes
Barbados cherry	48	*L. lactis*	*L. lactis*	yes
	49	*L. plantarum*	*L. plantarum*	yes
	50	*L. lactis*	*L. lactis*	yes
	53	*L. plantarum*	*L. plantarum*	yes
	54	*L. brevis*	*L. brevis*	yes
	55	*L. fermentum*	*L fermentum*	yes
	59	*L. brevis*	*L brevis*	yes
	60	*L. fermentum*	*L. fermentum*	yes
	61	*L. nagelii*	*L. nagelii*	yes
	138	*L. plantarum*	*L. plantarum*	yes
Soursop	104	*Lactobacillus* spp.	*L. fermentum*	yes
	106	*L. paracasei*	*L. casei*	no

	107	*Lactobacillus* spp.	*L. fermentum*	yes
	108	*L. paracasei*	*L. paracasei*	yes
	110	*L. nagelii*	*L. nagelii*	yes
	111	*L. fermentum*	*L. fermentum*	yes
	114	*L. nagelii*	*L. nagelli*	yes
	115	*L. nagelii*	*L. nagelli*	yes
	141	*L. fermentum*	*L. fermentum*	yes
	214	*L. nagelii*	*L. nagelii*	yes
Mango	128	*L. plantarum*	*L. plantarum*	yes
	129	*L. pentosus*	*L. pentosus*	yes
	133	*L. mesenteroides*	*L. mesenteroides*	yes
	136	*P. pentosaceus*	*P. pentosaceus*	yes
	139	*L. fermentum*	*L. fermentum*	yes
	149	*L. mesenteroides*	*L. mesenteroides*	yes
	198	*L. plantarum*	*L. plantarum*	yes
	199	*L. nagelii*	*L. nagelli*	yes
	201	*L. plantarum*	*L. plantarum*	yes
	205	*L. mesenteroides*	*L. mesenteroides*	yes
Strawberry	63	*L. fermentum*	*L. fermentum*	yes
	140	*Lactobacillus* spp.	*L. fermentum*	yes
	219	*L. fermentum*	*L. fermentum*	yes
	281	*L. fermentum*	*L. fermentum*	yes
	202	*Lactobacillus* spp.	*L. fermentum*	yes
	222	*L. fermentum*	*L. fermentum*	yes
	284	*L. fermentum*	*L. fermentum*	yes
	291	*Lactobacillus* spp.	*L. fermentum*	yes
	296	*L. fermentum*	*L. fermentum*	yes
